# Vasculitis Leading to Gangrene: An Early Presentation in a Rheumatoid Arthritis Patient

**DOI:** 10.7759/cureus.34438

**Published:** 2023-01-31

**Authors:** Abhishek Vadher, Karthik Yeruva, Chitralekha Vora, Karthik Gangu, Swati Baraiya

**Affiliations:** 1 Internal Medicine, B. J. Medical College, Ahmedabad, IND; 2 Internal Medicine, Merit Health River Region Hospital, Vicksburg, USA; 3 Internal Medicine, B. J. Medical and Civil Hospital, Ahmedabad, IND; 4 Internal Medicine, University of Kansas Medical Center, Kansas City, USA; 5 Family Medicine, Bombay Hospital and Medical Research Center, Mumbai, IND

**Keywords:** amputation, rheumatoid, arthritis, gangrene, vasculitis

## Abstract

Vasculitis is a late complication in rheumatoid arthritis (RA) and is seen in RA patients with long-standing disease. Rheumatoid vasculitis affects small-to-medium-sized vessels. In a few patients, vasculitis develops early in the course of the disease. Here, we report the case of a 32-year-old female who presented with gangrene in the second and third digits of the right foot and gangrene in the second digit of the left foot. She was on hydroxychloroquine and methotrexate for one year since the diagnosis of RA. The patient then developed Raynaud’s phenomenon and blackish discoloration of toes. She was started on pulse methylprednisolone, aspirin, nifedipine, and pentoxifylline. As no improvement was seen, intravenous cyclophosphamide was started. There was no improvement even after starting cyclophosphamide, and the gangrene further worsened. Eventually, after consulting the surgical team, it was decided to amputate the digits. The second digits in both feet were subsequently amputated. Hence, a physician should always be careful in checking for signs of vasculitis in RA patients early in the course of the disease as well.

## Introduction

The most serious complication of rheumatoid arthritis (RA) is vasculitis, which has high morbidity and mortality. It remains a rare and challenging complication of RA. Vasculitis in RA generally develops in a patient with a long-standing history of RA. Vasculitis early in the onset of RA is very unusual. In rheumatoid vasculitis (RV), small and medium-sized vessels are affected, and it affects about 1-5% of RA patients [1]. Although it lacks specific signs and symptoms, a majority of clinical manifestations have a predilection for the skin, with peripheral ulcers and cutaneous gangrene being the most common manifestations. The peripheral nervous system is also commonly affected presenting as mononeuritis multiplex or polyneuritis multiplex. Due to the non-specific signs and symptoms, its diagnosis is challenging and primarily depends on excluding other causes of similar lesions, such as diabetes, atherosclerosis, infection, or neoplasia. Ideally, a diagnosis is made by histopathological examination of the affected tissue which shows necrotizing vasculitis. There are many promising new drugs for the treatment of RA, but no randomized controlled trials have tested any drug’s efficacy and safety profile. Hence, the treatment is largely empirical and depends on the clinician’s choice, experience with the drug, and availability of the drug. Although the incidence of RV has been decreasing over the last two decades, possibly due to early diagnosis of RA and a more refined management approach to RA, it remains one of the most challenging and important complications of RA and should be promptly recognized and treated [1].

## Case presentation

**Figure 1:**
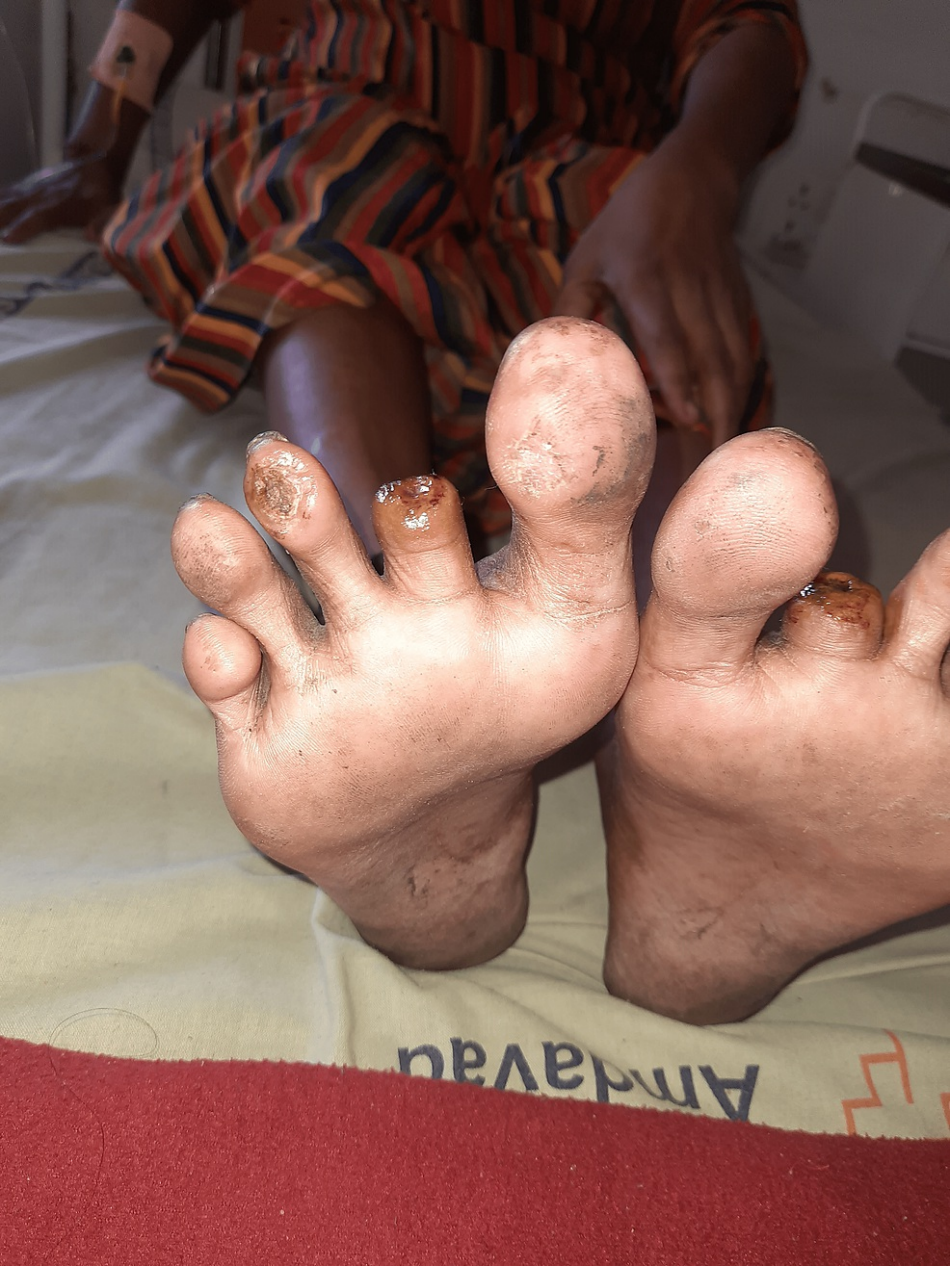
Post-amputation of the second digit of the right leg (ventral view).

**Figure 2:**
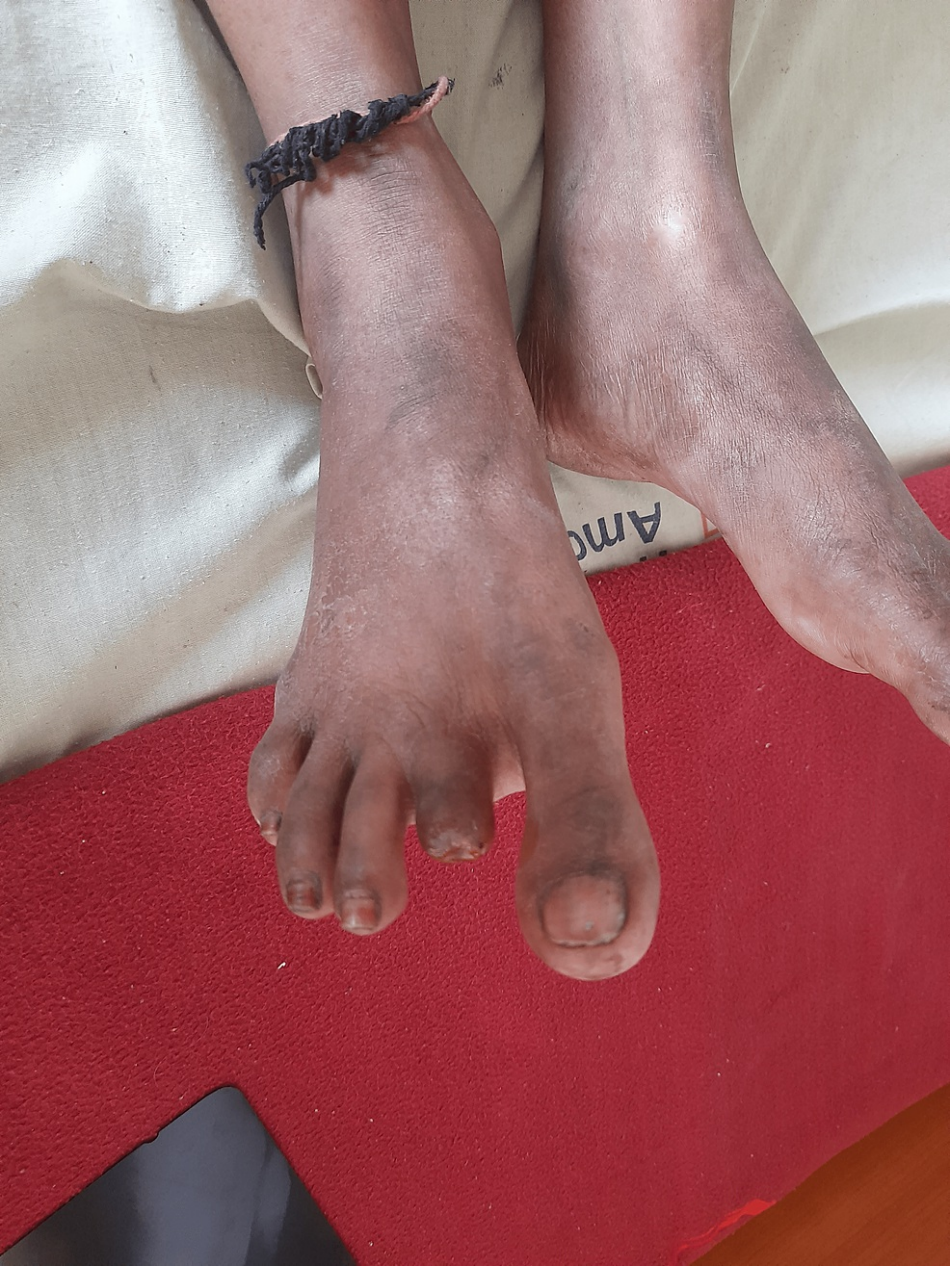
Post-amputation of the second digit of the right leg (dorsal view).

**Figure 3:**
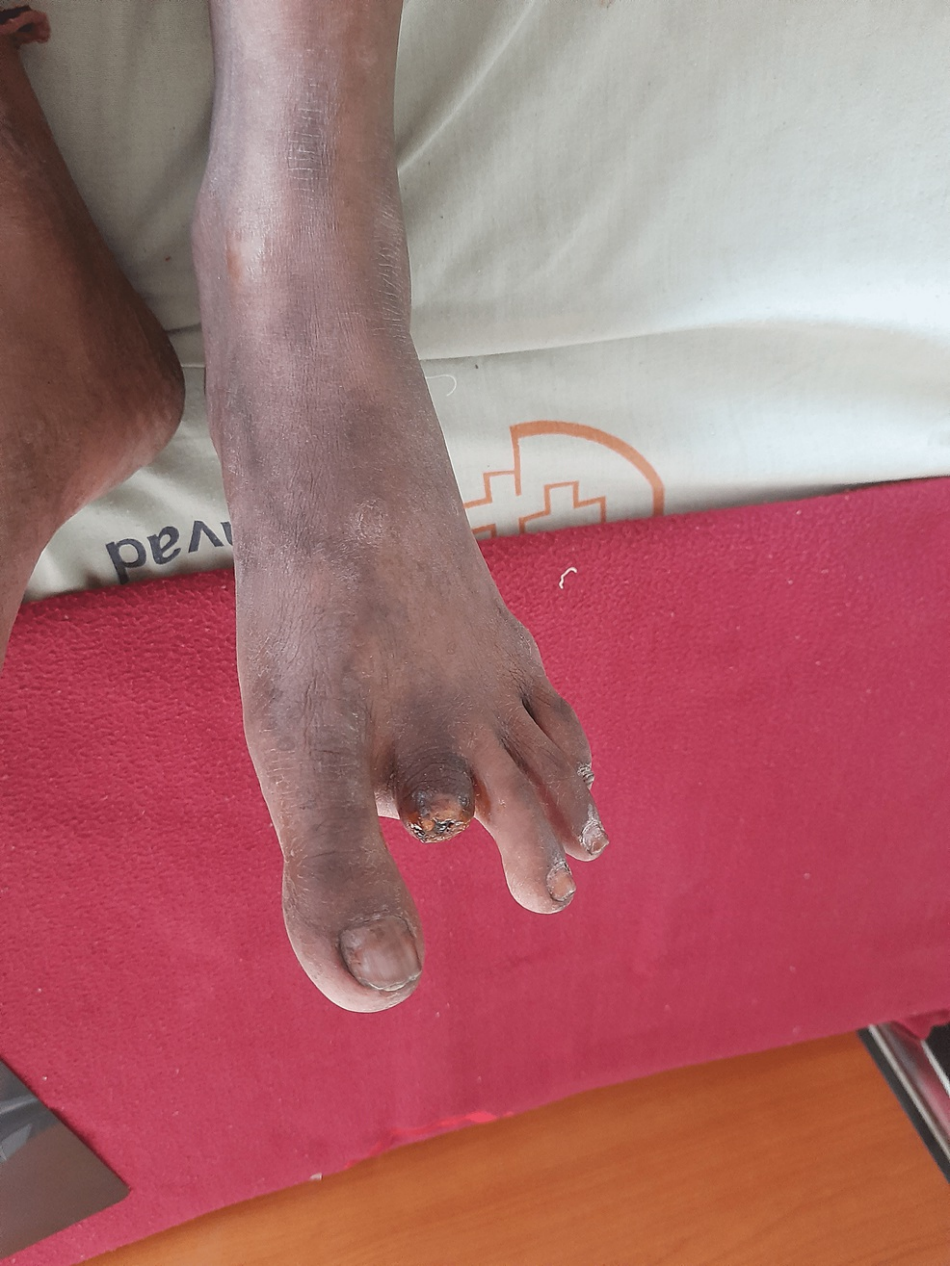
Post-amputation of the second digit of the left leg (dorsal view).

**Figure 4:**
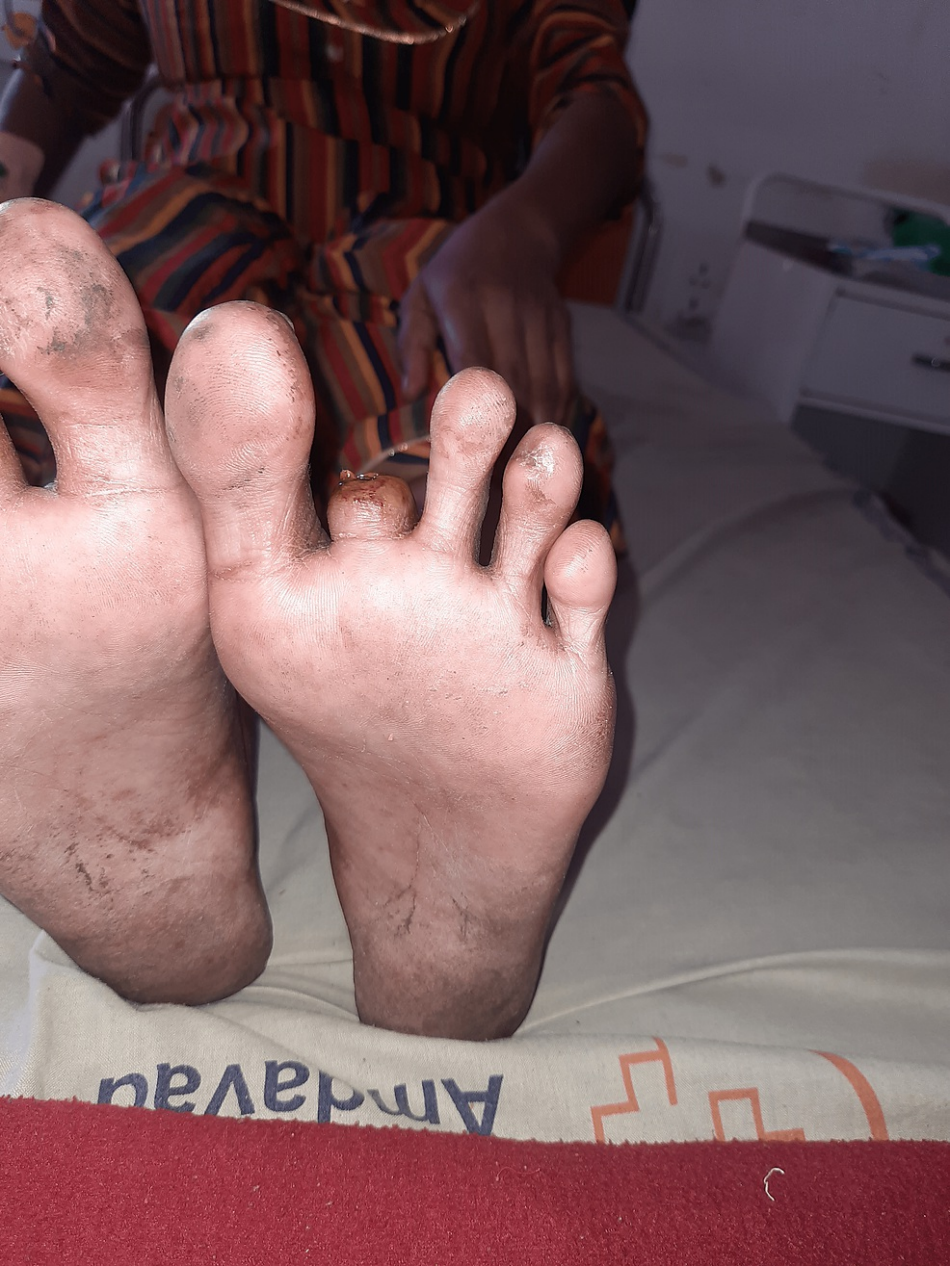
Post-amputation of the second digit of the left leg (ventral view).

A 32-year-old Indian female with a medical history of RA came to our hospital with a complaint of blackish discoloration of the second and third digits of the right foot and the second digit of the left foot. She was diagnosed with RA a year ago. At that time, she had a complaint of pain and tenderness in the metacarpophalangeal joints and proximal interphalangeal joints in both hands for about six months, along with morning stiffness for the same duration. She was started on tablet hydroxychloroquine 200 mg BD and tablet methotrexate 10 mg weekly with folinic acid. She did not have any other complaints apart from joint pain and stiffness a year back. Two months prior to the current hospitalization, she developed bluish discoloration of her index and middle fingers of both hands (Raynaud’s phenomenon) and noticed blackish discoloration of her toes one week before the current admission without fever, chills, or any other complaints.She was admitted to the Medicine Department and an urgent Surgical and Rheumatology consultation was done. She was started on pulse methylprednisolone 1 g/day for five days along with aspirin 75 mg, nifedipine 60 mg OD, and pentoxifylline 400 mg TID. Bilateral lower limb arterial and venous Doppler was normal. The supine resting ankle-brachial Index was 1.1 in both lower limbs. No improvement was seen after seven days. Subsequently, she was started on injection cyclophosphamide 500 mg intravenously which was given weekly for two weeks. There was no improvement after injection cyclophosphamide, and the gangrene increased in size. After one week, during a surgical consult, it was decided that the gangrene and necrosis were beyond recovery, and it was decided to amputate the second digits of the right leg (Figures 1, 2) and left leg (Figures 3, 4) and to observe the third digit of the right leg (Figure 1).

Investigations

The investigations showed hemoglobin of 9.3 g/dL (normal range = 11.5-15 g/dL), normal white blood cell count, and normal platelet count. The erythrocyte sedimentation rate (ESR) was 109 mm/hour (normal range = 0-29 mm/hour). Ferritin was 360 µg/L (normal range = 11-307 µg/L). C-reactive protein (CRP) was 44 mg/L (normal range <9 mg/L). The rheumatoid factor level was 45.4 IU/mL (normal range <15 IU/mL). Antinuclear antibody (ANA) titer was <1:32 and negative for ANA patterns. Human immunodeficiency virus antibodies and hepatitis panel were negative. Cytoplasmic antineutrophil cytoplasmic antibodies (c-ANCA) and perinuclear anti-neutrophil cytoplasmic antibodies (p-ANCA) were negative. X-ray of bilateral hands and feet showed no signs of erosion or joint space narrowing.

Treatment

The patient was started on pulse methylprednisolone 1 g/day for five days with aspirin 75 mg, nifedipine 60 mg OD, and pentoxifylline 400 mg TID. After no improvement for a week, the patient was given intravenous cyclophosphamide 500 mg weekly for two weeks. There was minimal improvement after treatment.

## Discussion

RV is a rare complication and is generally seen in patients with long-standing RA. Any organ of the body can be involved, and it generally affects small-to-medium-sized vessels. The mean duration between RA diagnosis and vasculitis development is 10-14 years. Very rarely it develops during the first five years of RA diagnosis [1]. Recently, there has been widespread use of biological agents early in the course of the disease, and this has led to a decrease in the prevalence of RV, as reported in the study by Bartels et al. [2,3].

RV is seen almost exclusively in seropositive nodular RA patients. RA is commonly seen in patients with *HLA-DRB1* mutations. A recent meta-analysis involving more than 1,500 RA patients showed that RV is common in three specific genotypes of the *HLA-DRB1* shared epitope, namely, *0401/*0401, *0401/*0404, and *0101/*0401 [3]. A strong association of smoking with the development of RV was also reported in the study by Mayo Clinic Rochester Epidemiology Project and many Swedish cohorts [4]. A new association was also found between *HLA-C3* with RV which was not related to the *HLA-DRB1* disequilibrium [4]. These studies showed that there is a heterogeneity of genetic and environmental factors in the pathogenesis of RV.

Many studies suggest that RV may be caused by circulating immune complexes containing rheumatoid factor (RF) and autoantibodies such as anti-endothelial cell antibodies, which form deposits in vessel walls and trigger an inflammatory reaction that leads to an injury of the endothelium [5]. Skin and peripheral nerves are the most common organs involved in RV, and the involvement of these organs is seen in more than 80% of the patients [6]. The heart, bowel, and kidneys are less commonly involved but may lead to myocardial infarction, bowel ischemia, and renal failure, respectively, which leads to significant morbidity and mortality [7]. Cutaneous manifestations such as palpable purpura, nodules, ulcers, nail fold infarctions, digital necrosis, livedo reticularis, and urticarial vasculitis are seen in RV.

The proposed diagnostic criteria for RV consist of one or more of the following features along with RA diagnosis in a patient [1]: peripheral gangrene [2], peripheral neuropathy, or mononeuritis multiplex. Biopsy shows acute necrotizing arteritis with fever and weight loss. Deep cutaneous ulcers or extra-articular disease (e.g., pleurisy, pericarditis, scleritis), if associated with biopsy, is evidence of vasculitis or typical digital infarcts [8].

There are no laboratory tests to confirm RV [3]. Laboratory tests only support the diagnosis of RV. An association between high titers of RA and anti-cyclic citrullinated peptide (anti-CCP) antibodies is found in RV [9]. An association between low complement levels and RV has also been found in 20-57% of patients in some studies [10].

Clinical approach

A high degree of suspicion is needed in a patient of RA with extra-articular manifestations and constitutional symptoms of weight loss and fever showing skin lesions or nervous system abnormalities. Biopsy remains the standard diagnostic test for definitive diagnosis. Skin biopsy has up to 75% yield in diagnosis. A biopsy can be directed by the electrophysiologic study of peripheral nerves showing subclinical defects [11]. Some studies recommend rectus femoris muscle biopsy while others recommend sural and peroneal nerve biopsy [11]. The diagnostic yield of muscle or nerve biopsy may be less than 50% in muscle or nerve biopsy. Angiographic findings are also non-specific and not generally used except in the setting of bowel ischemia [3].

After an initial diagnosis, a complete review of all systems should be done to determine the extent of the involvement in vasculitis. The treatment can be decided accordingly. Laboratory findings only support the diagnosis and do not help in confirming the diagnosis. Laboratory findings include anemia of chronic disease, ESR and CRP elevation, and high titer of RA antibodies and anti-CCP antibodies. Atypical p-ANCA antibodies can be found in 48% of patients with RV [12]. Hence, the diagnosis of RV depends on the combination of clinical findings and laboratory reports.

Management

The degree of organ system involvement determines the aggressiveness of the treatment. Mild RA involving skin or peripheral nerves is generally treated with prednisone and azathioprine or methotrexate. Higher doses of steroids with cyclophosphamide/biological agents are generally used for the treatment of more serious organ system involvement. Central nervous system involvement, acute renal failure, acute myocardial infarction, or ischemic bowel require treatment with intravenous steroids and consideration of cytotoxic or biological agents. In biological agents, anti-tumor necrosis factor (TNF) agents and rituximab are used.

A 2008 case series showed nine patients with refractory RV, i.e., prior failure with cyclophosphamide being treated with TNF inhibitors [13]. Seven patients successfully completed six months of treatment. Among these patients, five achieved complete remission, one achieved partial remission, and one failed remission. There are many case reports in the literature where anti-TNF therapy was used successfully to achieve remission [14-17].

Three cases have also been reported where rituximab was successfully used to treat RV [18,19]. Some reports also state a possible causal link between the use of rituximab and anti-TNF agents and RV.

Although RV is due to an inflammatory process, aggressive treatment of traditional risk factors of atherosclerosis, smoking cessation, and controlling dyslipidemia, hypertension, and diabetes are strongly recommended [20].

## Conclusions

Vasculitis is a late complication in RA and is seen in RA patients with long-standing disease. Rarely, it can be seen in patients with RA early in the course of the disease, such as in our patient. RV affects small-to-medium-sized vessels. It can present as gangrene in toes or fingers, such as the presentation in our patient, or it can present as an ulcer. Hence, a physician should always consider this possibility while treating RA patients.
